# P-260. Antibiotic residues in hospital sink p-trap water in Kilifi, Kenya

**DOI:** 10.1093/ofid/ofae631.464

**Published:** 2025-01-29

**Authors:** Lauren Hookham, Caroline Tigoi, Anne Amulele, Gillian Rodger, Kevin Chau, Robert Musyimi, James A Berkley, Nicole Stoesser

**Affiliations:** Modernising Medical Microbiology, Nuffield Department of Medicine, University of Oxford, Oxford UK, Oxford, England, United Kingdom; KEMRI/Wellcome Trust Research Programme, Kenya. KEMRI/Wellcome Trust Research Programme, Kenya., Nairobi, Nairobi Area, Kenya; KEMRI/Wellcome Trust Research Programme, Kenya. KEMRI/Wellcome Trust Research Programme, Kenya., Nairobi, Nairobi Area, Kenya; Nuffield Department of Medicine, University of Oxford, Oxford, UK Health Protection Research Unit in Healthcare Associated Infections and Antimicrobial Resistance, University of Oxford, Oxford, UK, Oxford, England, United Kingdom; Nuffield Department of Medicine, University of Oxford, Oxford, UK Health Protection Research Unit in Healthcare Associated Infections and Antimicrobial Resistance, University of Oxford, Oxford, UK, Oxford, England, United Kingdom; KEMRI/Wellcome Trust Research Programme, Kenya. KEMRI/Wellcome Trust Research Programme, Kenya., Nairobi, Nairobi Area, Kenya; Centre for Tropical Medicine & Global Health, University of Oxford & KEMRI/Wellcome Trust Research Programme, Kenya., Kilifi, Coast, Kenya; Oxford University, Oxford, England, United Kingdom

## Abstract

**Background:**

The hospital environment, including sink drains, may act as reservoirs for bacteria and antimicrobial resistance (AMR) gene amplification and spread. Selective pressures in sink environments, such as from disposal of antibiotics, may promote AMR. This pilot study at Kilifi County Hospital, Kenya, aimed to describe the prevalence of common antimicrobials in wastewater.

Presence of antibiotics in sinks by ward type

**Figure:**
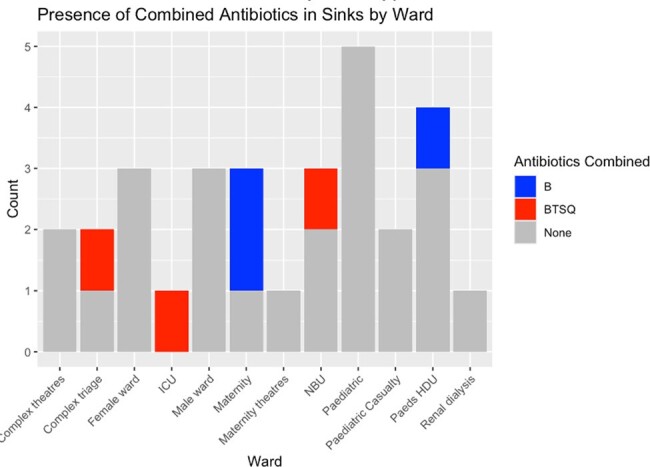

Presence of antibiotics (beta-lactam, sulpha, quinolone or tetracycline) detected in p-trap water of sinks in in-patient wards at Kilifi County Hospital, Kenya.

**Methods:**

Data were collected on the number of beds/patients/sinks per bay and ward, distance from sink to patient bed, and sink design. We aimed to obtain 50ml of p-trap water from different sink types (i.e. bay, sluice, toilet) across all hospital wards.

Dipstick testing was performed using the QuaTest BTSQ 4-1 kit, which was previously validated using serial dilutions of ampicillin, doxycycline, sulfamethoxazole and ciprofloxacin (1). All samples were processed at Kenya Medical Research Institute Wellcome Trust Research Programme on the day of collection.

**Results:**

34 sinks were targeted across nine wards. Two sinks on the neonatal birth unit (NBU) were broken or blocked and not sampled. One sink p-trap on NBU could not be aspirated despite running water to fill the p-trap. 1 sample from ICU was lost in transit from hospital to laboratory. In total, p-trap aspirates from 30 sinks were available for analysis.

Antibiotic testing failed in 1/30 (3%) samples, with absent control lines. Antibiotics were detected in 6/30 (20%) samples (complex triage, adult ICU, maternity, NBU, paediatric HDU). Most positive samples (67%, 4/6) were from patient bays, and the remainder from sluices (33% 2/6). No association was found between the presence of any antibiotic in p-trap water and building or sink type. The proportion of sinks in wards caring for pregnant women (50%, 2/4) and neonates (67%, 2/3) with detectable beta-lactam antibiotics was higher than wards caring for adults or children (p=0.03).

**Conclusion:**

We describe prevalence of antibiotic residue in hospital wards at Kilifi County Hospital, Kenya. Antibiotics were predominantly found in patient bays in wards caring for pregnant women and neonates. This finding may reflect differences in antibiotic usage and waste disposal practices. Targeted interventions with healthcare staff and hospital governance are necessary to promote responsible antibiotic and patient waste management.

**Disclosures:**

**All Authors**: No reported disclosures

